# Supplementary material for: Engineering induction of singular neural rosette emergence within hPSC-derived tissues
Source: eLife. 2018 Oct 29;7:e37549. doi: 10.7554/eLife.37549 (PMC6205811; doi:10.7554/eLife.37549)
Supplement: Supplementary file 1. — Table S1 and S2 provide summary statistics on human vs. algorithm (i.e. the automated neuroepithelial image analysis algorithm and machine learning classifier) error rates. Table S1 reports analysis conducted on 300 μm diameter (0.071 mm2) circular tissue images, and Table S2 reports analysis conducted on 35 neuroepithelial tissue images selected randomly and distributed across all morphologies. Table S3 and S4 provide catalog and usage information for primary antibodies and QPCR primer sets used in this study. [file elife-37549-supp1.docx]

**Supplemental Information**

**Engineering Induction of Singular Neural Rosette Emergence within hPSC-derived Tissues**

Gavin T. Knight, Brady F. Lundin, Nisha Iyer, Lydia M.T. Ashton, William A. Sethares, Rebecca M. Willet, Randolph S. Ashton

Inventory of Supplemental Information

Table S1

Table S2

Table S3

Table S4

**Table S1. Comparison of human vs. machine learning classifier analysis of 300μm circular neuroepithelial tissues. (**Figure 2-Source Data)

|  | **Error** | **Stdev** | **False +** | **Stdev** | **False -** | **Stdev** |
| --- | --- | --- | --- | --- | --- | --- |
| **Polarization** |  |  |  |  |  |  |
| Human | 21.19% | 3.35% | 13.56% | 6.94% | 7.63% | 3.81% |
| Machine Learning | 14.35% |  | 7.59% |  | 6.75% |  |
| **Rosette** |  |  |  |  |  |  |
| Human | 13.05% | 2.74% | 9.15% | 4.06% | 3.90% | 1.51% |
| Machine Learning | 8.86% |  | 3.38% |  | 5.49% |  |

**Table S2. Comparison of human vs. machine learning classifier analysis of neuroepithelial tissues with variable morphologies**. **(**Figure 2-Source Data)

|  | **Error** | **Stdev** | **False +** | **Stdev** | **False -** | **Stdev** |
| --- | --- | --- | --- | --- | --- | --- |
| **Polarization** |  |  |  |  |  |  |
| Human | 19.05% | 14.61% | 16.19% | 16.07% | 1.63% | 1.77% |
| Machine Learning | 20.27% |  | 12.16% |  | 8.11% |  |
| **Rosette** |  |  |  |  |  |  |
| Human | 9.93% | 6.96% | 9.39% | 7.40% | 0.54% | 0.75% |
| Machine Learning | 19.59% |  | 16.89% |  | 2.70% |  |

**Table S3. Primary Antibody List**.

| **Antigen** | **Host species** | **Clone or product #** | **Dilution** | **Vendor** |
| --- | --- | --- | --- | --- |
| Pax6 | Mouse | N/A | 1:200 (FC) | DSHB |
| Pax6 | Rabbit | PRB-278P | 1:500 (ICC) | Covance |
| Pax6 | Rabbit | 901301 | 1:200 (ICC) | BioLegend |
| N-cadherin | Mouse | 610920 | 1:500 (ICC) | BD Biosciences |
| Otx2 | Goat | AF1979 | 1:500 (ICC) | R&D Systems |
| βIII-tubulin/Tuj 1 | Rabbit | PRB-435P | 1:1000 (ICC) | Covance |
| Nkx6.1 | Goat | AF5857 | 1:200 (ICC) | R&D Systems |
| Olig2 | Rabbit | AB9610 | 1:200 (ICC) | Millipore |
| Phospho-Histone 3 | Rabbit | Ab10543 | 1:200 (ICC) | Abcam |
| ZO-1 | Mouse | 610967 | 1:200 (ICC) | BD Biosciences |
| Phalloidin-TRITC | NA | P1951 | 1:200 (ICC) | Sigma |

**Table S4. TaqMan Primer Set List.**

| **Gene** | **Life Technologies Assay ID** |
| --- | --- |
| *RPS18* | Hs01375212_g1 |
| *HOXA2* | Hs00534579_m1 |
| *HOXB4* | Hs00256884_m1 |
| *HOXC6* | Hs00171690_m1 |
| *HOXC9* | Hs00396786_m1 |
| *HOXD10* | Hs00157974_m1 |
| *Pax6* | Hs01088114_m1 |
| *Otx2* | Hs00222238_m1 |
| *FoxG1* | Hs01850784_s1 |
